# Maternal Transmission of Rotavirus to Calves and Comparison of Colostrum and Fecal Microbiota in Holstein and Hanwoo Cattle

**DOI:** 10.3390/vetsci11120606

**Published:** 2024-11-28

**Authors:** Seon-Ho Kim, Michelle Miguel, Ye Pyae Naing, Yong-Il Cho, Sang-Suk Lee

**Affiliations:** 1 Ruminant Nutrition and Anaerobe Laboratory, Department of Animal Science and Technology, Sunchon National University, Suncheon 57922, Republic of Korea; mamiguel@scnu.ac.kr (M.M.); 1205047@s.scnu.ac.kr (Y.P.N.); 2 Animal Disease and Diagnostic Laboratory, Department of Animal Science and Technology, Sunchon National University, Suncheon 57922, Republic of Korea; ycho@scnu.ac.kr

**Keywords:** calf, cow, Hanwoo, Holstein, maternal transmission, rotavirus

## Abstract

This study aimed to evaluate rotavirus transmission to calves and analyze microbial communities in cow milk and neonatal calf feces within dairy and beef cattle. A total of 20 cattle, Hanwoo (*n* = 10), and Holstein (*n* = 10) were allotted for the study, with each breed comprising five cows and five calves. Colostrum samples were obtained from the dam, while feces were obtained from both the dam and calf. Group A rotavirus was identified in the fecal samples through real-time reverse transcription PCR (RT-qPCR). Bacterial communities present in the colostrum and bovine feces were explored using 16S rRNA metagenomic sequencing. The RT-qPCR results showed that the Cq value of one calf and one cow in the Holstein group was < 35, confirming the presence of rotavirus, whereas the Cq value in the Hanwoo group was > 35, indicating a negative result. For the bacterial communities, significant differences (*p* < 0.05) were found between the colostrum and fecal samples from the dams and calves, but there were no significant differences between Hanwoo and Holstein cattle. Alpha diversity analysis showed that the Chao1 and Shannon indices revealed significant differences (*p* < 0.05) among the sample types (cow colostrum, cow feces, and calf feces). The bacterial communities in various sample types from both Hanwoo and Holstein cattle were dominated by the phyla Firmicutes, Proteobacteria, and Bacteroidetes. In addition, the genera shared between the cow colostrum and calf fecal microbiota were higher than those shared between cow and calf feces. Overall, the current study detected rotavirus in Holstein but not in Hanwoo cattle; however, no clear evidence showed the transmission of rotavirus from dam to calf. Moreover, significant variations in bacterial compositions were observed among calf feces, cow feces, and colostrum samples, suggesting the presence of unique microbial profiles.

## Simple Summary

This study investigated the maternal transmission of rotavirus and compared the fecal and colostrum microbiota of Holstein and Hanwoo cattle raised in South Korea. A total of 20 cattle (ten Holstein and ten Hanwoo) were included in the study, with samples collected from both cows and calves. Group A rotavirus was detected in one calf and one cow from the Holstein group; however, no rotavirus was found in the Hanwoo group. The microbial diversity analysis showed significant differences in the bacterial composition of fecal and colostrum samples from both cows and calves. The study concludes that maternal microbial transfer plays a key role in shaping neonatal gut microbiota. Although rotavirus transmission was not conclusively demonstrated, the findings highlight breed-related microbial differences that may impact calf health and productivity.

## 1. Introduction

Neonatal calf diarrhea (NCD) is a condition that significantly affects newborn calves, typically within the first month of life. It is characterized by frequent, watery, and irregular bowel movements and is a leading cause of morbidity and mortality in calves [1,2,3]. Calf diarrhea is primarily caused by infectious agents, including viruses, bacteria, and protozoa. The key pathogens responsible for the condition are bovine rotavirus Group A (BRoV-A), bovine coronavirus (BCoV), *Salmonella* spp., *Escherichia coli*, *Clostridium perfringens* type C, and *Cryptosporidium parvum* [1,4]. In many cases, diarrhea has multiple contributing factors, with various enteropathogens often involved simultaneously in the condition. Rotavirus, a leading cause of neonatal diarrhea in calves, has a significant impact on calf health and cattle industry economics [5]. The virus is highly contagious, transmitted primarily via the fecal–oral route, and leads to significant economic losses due to reduced weight gain, treatment costs, and calf mortality [5,6]. The early immune response of calves, particularly in the first few days of life, is critically shaped by their intake of colostrum, which is rich in immunoglobulins, growth factors, and maternal microbiota [7].

The gut microbiota serves a protective role against pathogens; however, dysbiosis of the intestinal microbiota can impact various microbial functions, including those related to nucleotide transport and metabolism, defense mechanisms, and translation and transcription processes [2]. In addition, the gut microbiota plays a vital role in regulating host intestinal development, immune response, growth, and energy homeostasis [8]. In calves, the gut microbiota is influenced or changed by a multitude of factors, including the influence of their dams, genetics, environmental conditions, diet, and feed supplements [9,10]. Recent evidence suggests that maternal factors, including the transfer of microbial communities from the dam to offspring, play a vital role in shaping the gut microbiota [11]. Understanding the maternal influences on microbial communities in neonatal calves has important implications for optimizing the health and productivity of dairy and beef production systems. Several studies have highlighted the importance of maternal factors in shaping the gut microbiota during early life stages, emphasizing the vertical transmission of microbial communities from the dam to offspring [12,13,14,15,16].

The dam is a crucial source of microbes for the calf; therefore, variations in the development of the calf’s microbiota could be attributed to the farming system, which influences the extent of contact between the calf and the dam [14]. Bovine colostrum contains a diverse microbial community, which is transferred from the mother to the newborn through the ingestion of colostrum [17,18,19]. Exposure to microbes from the dam’s feces and vagina during birth initiates the colonization of the newborn’s gastrointestinal tract, which is further enhanced by the transfer of maternal microbes through licking and feeding from the dam [18,20,21]. The vertical transfer of maternal microbiota to newborns is well-documented across numerous animal species [17,18,22]. Research on dairy cattle has shown notable similarities between the neonatal gut microbiota and the microbial communities found in the dam’s feces, vagina, teat skin, saliva, and colostrum [17,18,20,21]. In cow–calf suckler systems, beef calves remain with the dam until weaning, promoting natural microbial transmission. In contrast, dairy farming practices often involve separating calves from the dam shortly after birth, housing them separately, and feeding them artificial milk replacers, limiting direct maternal microbial exposure [8,14,17]. In dairy production systems, the immediate separation of calves from their dams can potentially limit the vertical transfer of microbes [14]. Additionally, research has shown differences in the diversity of the rumen microbiome between goat kids raised apart from their dams and those raised alongside them [23].

Holstein and Korean native cattle (Hanwoo), two widely recognized cattle breeds, have distinct characteristics and performance traits. Holsteins are known for their high milk production capacity [24], while Hanwoo cattle exhibit excellent meat quality [25,26]. These breed-specific traits may be influenced by their gut microbiota composition, which can be modulated by the maternal microbial transfer. Maternal influences on the calves’ fecal microbiota, mediated through milk, can provide valuable insights into microbial transmission, establishment, and the subsequent impact on calf health and performance. However, the extent to which maternal factors contribute to the development of the gut microbiota in these two breeds remains largely unexplored.

Several studies have explored the relationship between colostrum feeding and the intestinal colonization of calves [19,21,27,28]; however, understanding the impact of maternal influence on the composition of fecal microbiota in these two prominent cattle breeds can provide valuable knowledge for optimizing management strategies in livestock production in Korea. The composition of the microbiota in colostrum and feces may differ based on cattle breeds, potentially influencing neonatal immunity and pathogen resistance. It may also contribute to the development of targeted interventions aimed at enhancing gut health and promoting optimal growth and performance in calves. Additionally, by characterizing and comparing the fecal microbiota of Holstein and Hanwoo cows and calves, we can gain insights into the breed-specific differences and identify potential microbial markers associated with desirable phenotypic traits. Hence, this study investigated the maternal transmission of rotavirus and characterized the microbial composition of colostrum and cow and calf feces in Holstein and Hanwoo cattle breeds. In addition, it examined the potential transfer of microbial communities from the dam to the calf and assessed the degree of maternal influence on the calf’s microbiota.

## 2. Materials and Methods

### 2.1. Animals and Sample Collection

Two breeds of cattle, Holstein (*n* = 10) and Hanwoo (*n* = 10) were used in the study, with each breed consisting of 5 cows and 5 calves. The Holstein and Hanwoo cattle were housed in separate farms. The Holstein and Hanwoo dams had an average parturition of 2.0 and 1.6, respectively. The Hanwoo cattle were fed with 5 kg of concentrate and ad libitum rice straw, whereas the Holstein were fed a total mixed ration.

Samples of colostrum (50 mL) were collected immediately after calving, within 24 h postpartum. Fecal samples (10 g) were taken from the dams after parturition and from the calves within the first 48 h after birth. The samples were placed in sterile containers and stored at −80 °C until further analysis.

### 2.2. Real-Time RT-PCR Assay Rotavirus Detection Detection of Bovine Rotavirus

RNA was extracted from the fecal samples using a commercially available kit (RNeasy^®^ Mini Kit, Qiagen, Hilden, Germany), following the manufacturer’s instructions. RT-qPCR was used to detect the presence of Group A rotavirus in the fecal samples using primers and probe specific for VP6 [29]. The primers and specific probe used were as follows: RVA-F: 5′–ACT CCA ATG TAA GTG ATC TAA TTC–3′, RVA-R: 5′–GAG TTG TTC CAA GTA ATC CAA A–3′, and RVA-P: 5′–FAM-ACC AAT TCC TCC AGT TTG GAA YTC ATT YCC-BHQ1–3′. The reaction volume was 20 µL, which included 10 µL of Hyperscript^TM^ One-step 2X RT-PCR Master mix (GeneAll Biotechnology Co., Ltd., Seoul, Republic of Korea), 1 µL of each primer and probe (10 pmol/µL), 2 µL of RNA template, and 5 µL of nuclease-free water. The thermal cycling conditions consisted of reverse transcription at 50 °C for 30 min, followed by an initial denaturation at 94 °C for 4 min. This was followed by 40 cycles of denaturation at 94 °C for 30 s, annealing at 55 °C for 1 min, and extension at 72 °C for 1 min, with a final extension at 72 °C for 7 min. Amplification was carried out using the CFX96 Touch Real-Time PCR Detection System (Bio-Rad, Hercules, CA, USA). For the standard curve calibration, a bovine rotavirus standard concentration of 61.8 ng/μL was used, and an 8-point 10-fold serial dilution curve was prepared. Nuclease-free water served as the no-template (negative) control (NTC). RT-qPCR amplification was performed using an optimized protocol on a CFX96 Touch Real-Time PCR Detection System (Bio-Rad). The standard control with the lowest concentration had a Cq of 35, while the no-template control (NTC) had a Cq of >35, suggesting negligible or no amplification; thus, a threshold of 35 was established as the Cq cut-off for positivity. Samples with Cq ≤35 were considered positive and samples with Cq >35 were considered negative.

### 2.3. Library Construction

Colostrum and fecal samples were sent to Macrogen (Macrogen Inc., Seoul, Republic of Korea) for metagenomic sequencing analysis. The microbiota of colostrum and feces were analyzed by amplifying the hypervariable V3–V4 region of the 16S rRNA gene, following the protocol outlined in the 16S Metagenomics Library Prep Guide (15044223 Rev. B). The sequencing was performed on the Illumina MiSeq platform with 2 × 300 bp paired-end reads (Illumina, Inc., San Diego, CA, USA), according to the manufacturer’s instructions.

### 2.4. Bioinformatics

The raw sequence data were processed using Quantitative Insights Into Microbial Ecology 2 (QIIME2) 2021.4 [30]. The paired-end reads obtained for each sample were demultiplexed to attribute sequence reads to the appropriate samples, then joined. The Divisive Amplicon Denoising Algorithm 2 (DADA2) plugin was used to truncate and trim the low-quality base calls of demultiplexed reads [31]. After quality control, the sequence reads were denoised and dereplicated into amplicon sequence variants (ASVs). Taxonomy was assigned to ASV representative sequences using BLAST+ (v.2.9.0) against the National Center for Biotechnology Information (NCBI) nucleotide and taxonomy databases [32].

All subsequent analyses were performed with R version 4.3.2 (R Core Team, Vienna, Austria) and R Studio 2024.09.0+375. The ASV count table, taxonomy, and metadata were imported into R using the phyloseq package [33]. Data were filtered for a minimum count of 4 with prevalence in 10% of samples. Alpha diversity was estimated by the Chao1 and the Shannon index. The Chao 1 index was used to estimate the species richness and the Shannon index was used to estimate species diversity. Beta diversity was assessed using the Bray–Curtis dissimilarity and visualized as a principal coordinate analysis (PCoA) plot. Taxonomic compositions at the phylum, genus, and species levels were represented using stacked bar graphs. Linear discriminant analysis (LDA) effect size (LEfSe) was used to identify significant genera that differed between the sample types (cow colostrum, cow feces, and calf feces) and the two breeds of cattle (Hanwoo and Holstein) [34]. The UpSet plot was generated with the R package UpSetR [35] and the Venn diagram was constructed using the jvenn tool to display the common and unique genera among the samples [36].

### 2.5. Statistical Analysis

Statistical differences in the alpha diversity index among different sample types within each breed were determined using the non-parametric Kruskal–Wallis test. Beta diversity differences were analyzed with permutational multivariate ANOVA (PERMANOVA) on 9999 permutations using the adonis2 function in the vegan package of the R software [37]. PERMDISP was used to assess variations in beta diversity by measuring the average distances of communities to their respective group of multivariate centroids [38]. PERMDISP was performed using the betadisper function in the vegan package of R. Statistical differences with *p*-values less than 0.05 were considered significant. For the LEfSe analysis, the conditions were established as follows: an alpha value of less than 0.05 for the Kruskal–Wallis test among classes, an alpha value of less than 0.05 for the pairwise Wilcoxon test among subclasses, and an LDA score (log 10) > 2.

## 3. Results

### 3.1. Detection of Rotavirus in Feces

**Figure 1:**
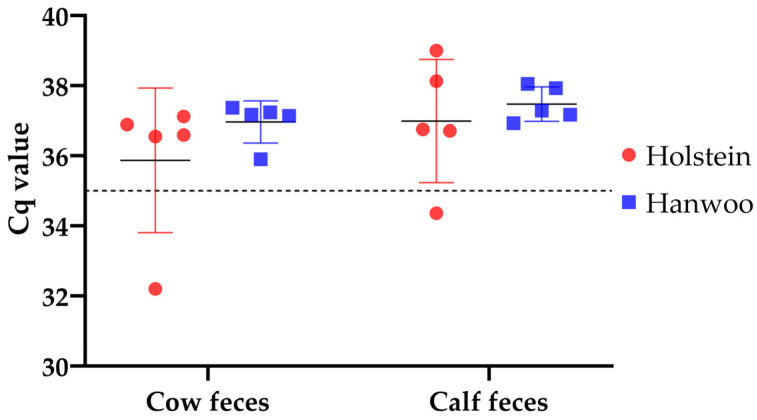
Amplification of bovine rotavirus Group A from Holstein and Hanwoo cow and calf fecal samples. A Cq cut-off value of 35 was considered positive.

The RT-qPCR results revealed that the Cq values of one calf and one cow from the Holstein group were less than 35, indicating the presence of rotavirus, while the Cq values in the Hanwoo group were greater than 35, suggesting a negative result, as depicted in Figure 1.

### 3.2. Diversity Analysis of Colostrum and Fecal Microbiota

**Figure 2:**
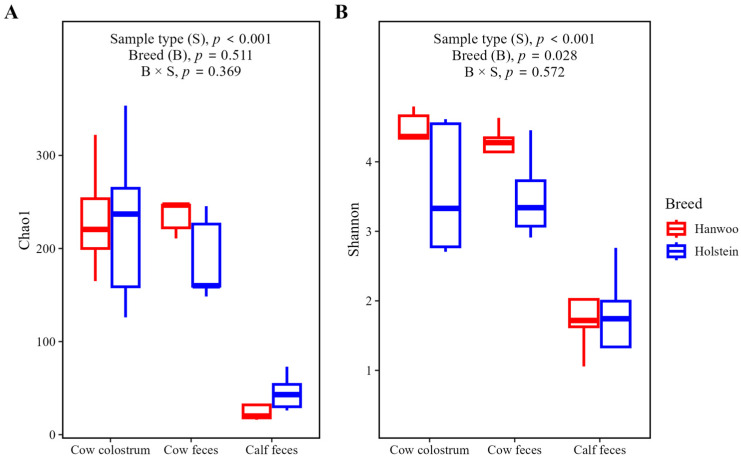
Alpha diversity of bacterial communities in cow (colostrum and feces) and calf (feces) samples in Hanwoo and Holstein breeds. Boxplots for Chao1 richness index (**A**) and Shannon diversity index (**B**).

The results of the alpha diversity analysis revealed significant differences (*p* < 0.05) in the Chao1 and Shannon indices between sample types (cow colostrum, cow feces, calf feces). Breed had no significant effect on the Chao1 index, but had significant effects on the Shannon index, as demonstrated in Figure 2.

**Figure 3:**
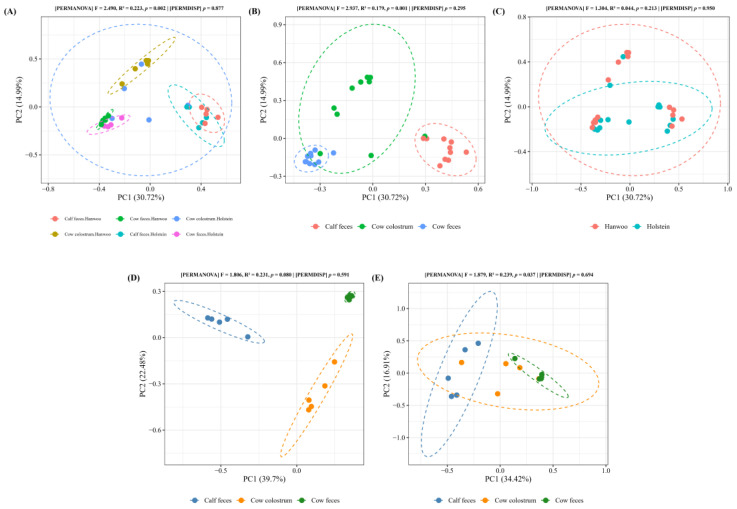
Principal coordinate analysis (PCoA) plot based on the Bray–Curtis dissimilarity in beta diversity based on sample type and breed (**A**), sample type (cow colostrum, cow feces, and calf feces) (**B**), breed (Hanwoo and Holstein) (**C**), different sample types within the Hanwoo group (**D**), and different sample types within the Holstein group (**E**). The *p*-value corresponds to results from the adonis PERMANOVA.

Bray–Curtis distances were calculated and represented as a principal coordinate analysis (PCoA) (Figure 3). Beta-diversity analysis revealed dissimilarities between the bacterial compositions of cow and calf samples in each breed (Figure 3A) and across sample types (cow colostrum, cow feces, and calf feces) (Figure 3B) (PERMANOVA, *p* < 0.05). The non-significant PERMDISP test confirmed that the significant differences identified by the PERMANOVA test were due to the variations in community structure between sample types rather than unequal dispersions of variance (PERMDISP, *p* > 0.05).

No differences were found between the bacterial communities in the Hanwoo and Holstein breeds (*p* > 0.05), as shown in Figure 3C. Similarly, there were no significant variations among the bacterial communities in different sample types (cow colostrum, cow feces, and calf feces) from Hanwoo cattle (*p* > 0.05) (Figure 3D). In contrast, significant differences (*p* < 0.05) were noted in the bacterial communities of different sample types in Holstein cattle (Figure 3E).

### 3.3. Shared Genera Between Cow Colostrum and Fecal Microbiota in Hanwoo and Holstein

**Figure 4:**
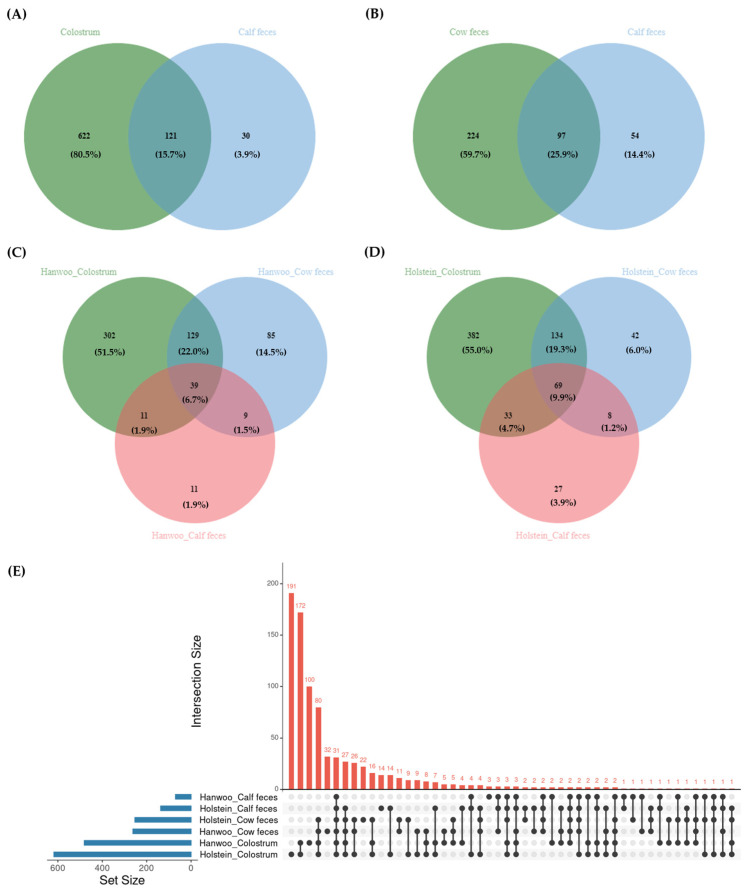
Venn diagrams showing the genera shared between cow colostrum and calf feces (**A**), cow feces and calf feces (**B**), different cow and calf samples in Hanwoo (**C**) and Holstein cattle (**D**), and (**E**) UpSet diagram showing genus distribution across sample types in different breeds of cattle. The bars represent the number of shared genera between specific samples or those unique to a single sample.

A total of 121 (15.7%) genera were found to be common between cow colostrum and calf fecal microbiota (Figure 4A), and 97 (25.9%) genera were shared between the fecal microbiota of cows and calves (Figure 4B). In Hanwoo cattle, a total of 11 (1.9%) genera were exclusively present in the cow colostrum and calf fecal microbiota, while 9 (1.5%) genera were common to both cow and calf fecal microbiota (Figure 4C). In Holstein, 33 (4.7%) genera were common in cow colostrum and calf fecal microbiota, whereas eight (1.2%) genera were shared between the fecal microbiota of cows and calves (Figure 4D). Figure 4E exhibits the distinct clustering patterns across all samples, with the variations observed in the microbiome within each sample type and among different breeds. The colostrum microbiota of Holstein cattle has a greater number of genera compared to the other samples.

### 3.4. Composition of Colostrum and Fecal Microbiota

**Figure 5:**
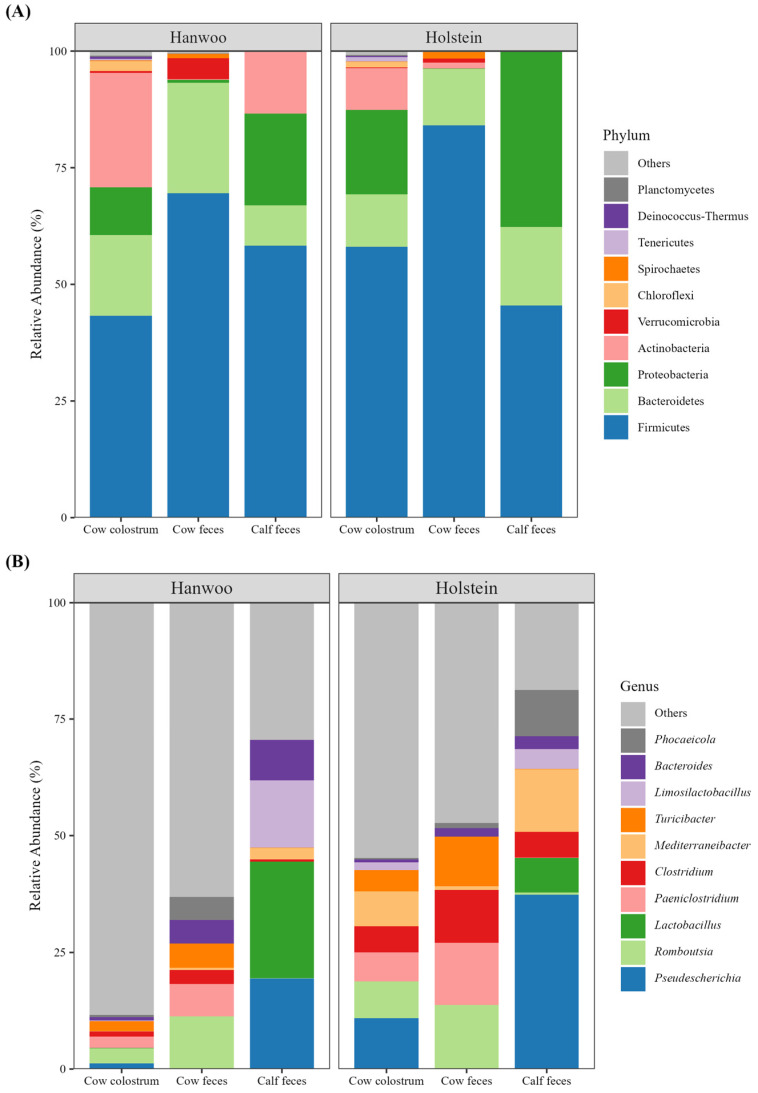
Taxonomic composition of bacterial communities in the colostrum and feces of Hanwoo and Holstein cows and calves. Relative abundance at phylum (**A**) and genus (**B**) levels.

Taxonomic compositions of bacterial communities in cow colostrum, cow feces, and calf feces in the Hanwoo and Holstein breeds are presented in Figure 5. Firmicutes, Proteobacteria, and Bacteroidetes were the most prevalent phyla identified in the bacterial communities of different sample types from Hanwoo and Holstein cattle (Figure 5A). Colostrum samples were dominated by the phyla Firmicutes, Bacteroidetes, Proteobacteria, and Actinobacteria, while Firmicutes, Bacteroidetes, and Proteobacteria were the most abundant phyla in the cow and calf fecal samples.

In the cow colostrum, the relative abundance of Firmicutes and Proteobacteria was higher in Holstein cattle than compared to Hanwoo cattle. In contrast, the relative abundance of Bacteroidetes and Actinobacteria was higher in Hanwoo cattle than in Holstein cattle. In the cow feces, Firmicutes was lower and Bacteroidetes was higher in Hanwoo cattle than in Holstein cattle. In the calf feces, Firmicutes and Actinobacteria had a higher relative abundance in Hanwoo cattle, while Bacteoidetes and Proteobacteria had a higher relative abundance in Holstein cattle compared to Hanwoo cattle.

At the genus level, the top ten most abundant genera identified across the samples included *Pseudescherichia*, *Romboutsia*, *Lactobacillus*, *Paeniclostridium*, *Clostridium*, *Mediterraneibacter*, *Turicibacter*, *Limosilactobacillus*, *Bacteroides*, and *Phocaeicola* (Figure 5B). In the cow colostrum, *Pseudescherichia*, *Romboutsia*, and *Mediterraneibacter* were the most abundant genera, while in cow feces, *Romboutsia*, *Paeniclostridium*, *Clostridium*, and *Turicibacter* were the most prevalent bacterial genera. On the other hand, *Pseudescherichia*, *Lactobacillus*, *Limosilactobacillus*, *Clostridium*, and *Bacteroides* were among the most prevalent genera found in the calf feces.

### 3.5. Identification of Significantly Abundant Colostrum and Fecal Bacterial Taxa in Hanwoo and Holstein Cattle

**Figure 6:**
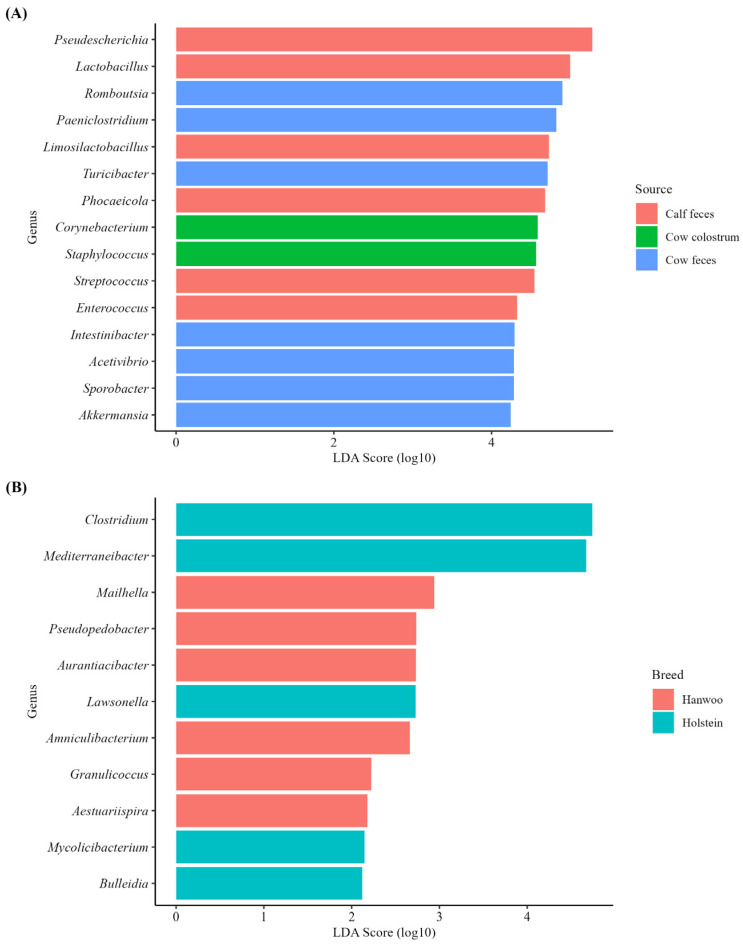
Differential abundance analysis of the bacterial genera in different sample types (cow colostrum, cow feces, and calf feces) (**A**) and breed (Hanwoo and Holstein) (**B**).

The bacterial genera identified by LEfSe analysis to be significantly differently represented among the three sample types and between cattle breeds are shown in Figure 6. The top 15 genera found to be differentially abundant across various sample types are presented in Figure 6A. Specifically, two genera were more abundant in cow colostrum, six were more abundant in cow feces, and seven were more abundant in calf feces. The bacterial genera *Corynebacterium* and *Staphylococcus* were significantly more abundant in cow colostrum, *Romboutsia*, *Paeniclostridium*, *Turicibacter*, *Intestinibacter*, *Acetivibrio*, *Sporobacter*, *and Akkermansia* were more abundant in cow feces, and *Pseudescherichia*, *Lactobacillus*, *Limosilactobacillus*, *Phocaeicola*, *Streptococcus*, and *Enterococcus* were significantly more abundant in the calf feces; (Figure 6A). Meanwhile, the 11 genera that were shown to be differentially abundant between Hanwoo and Holstein breeds are presented in Figure 6B. In the Holstein breed, the bacterial genera *Clostridium*, *Mediterrneibacter*, *Lawsonella*, *Mycolicibacterium*, and *Bulleidia* were enriched, while *Mailhella*, *Pseudopedobacter*, *Aurantiacibacter*, *Amniculibacterium*, *Granulicoccus*, and *Aestuariispira* were found to be enriched in Hanwoo cattle.

## 4. Discussion

In the present study, we investigated the transmission of rotavirus and microbiota between calves and their dams in two distinct cattle breeds, Hanwoo and Holstein. Rotavirus was detected in one cow and one calf in the Holstein group; however, no direct transmission from cow to calf was observed. This suggests that other factors, such as environmental contamination or fecal–oral transmission, may have contributed to the rotavirus-associated diarrhea. In contrast, all samples from the Hanwoo group tested negative for rotavirus, suggesting possible breed-related susceptibility, with Holstein cattle potentially being more vulnerable to infection. However, due to the limited sample size, further research is required to confirm these breed-specific differences. In addition to vertical transmission from dam to calf, rotavirus primarily spreads via fecal–oral transmission, with virus particles being shed in the feces of infected animals [5,39]. Contaminated feed, water, bedding, and equipment serve as significant sources of rotavirus infection [40]. Additionally, environmental factors such as poor sanitation, overcrowding, and shared housing further facilitate viral spread by increasing the risk of exposure to contaminated materials.

Neonatal calf diarrhea is a multifactorial condition which leads to high neonatal morbidity and mortality and is often associated with imbalances in gut microbiota [1]. According to Li et al., dysbiosis, characterized by a low-diversity microbiome, is a key factor in promoting diarrhea in neonatal calves [2]. Proper intake of colostrum and milk is crucial for gut health and the development of the calf’s immune system [19]. When dysbiosis occurs, it can lead to digestive diseases such as diarrhea and intestinal inflammation [10].

In this study, diversity analysis revealed significant differences in bacterial diversity between cow colostrum, cow feces, and calf feces but no notable differences between the two cattle breeds. Alpha diversity analysis, which assessed species richness (Chao1 index) and species diversity (Shannon index), indicated notable variations among sample types; however, differences in species diversity were also observed between the two breeds. This suggests that the microbial environments in colostrum, cow feces, and calf feces differ significantly within individual cows and their offspring, but the two breeds themselves do not have stark differences in their microbial diversity. Furthermore, we observed that the colostrum sample from the Holstein breed contained a higher number of genera, and the feces of Hanwoo calves had the lowest number of genera. This suggests a higher microbial diversity in Holstein colostrum compared to other sample types, while calf feces (especially in Hanwoo cattle) may harbor a more specialized or less diverse microbial community. In addition, the higher microbial diversity in Holstein colostrum may point to differences in diet, management practices, or physiological traits between breeds.

Beta diversity analysis further supported the significant differences in microbial composition between sample types, which can be attributed to the distinct ecological niches of colostrum, cow feces, and calf feces. However, no significant differences in beta diversity were observed between Hanwoo and Holstein breeds, which suggests that maternal influence and environmental factors might play a more prominent role in shaping the calf gut microbiota than breed alone. Meanwhile, a statistical trend was noted in the beta diversity among the different sample types within the Hanwoo group. Although the differences observed in Figure 3D did not reach statistical significance (*p* = 0.077), the trend observed suggests potential biological relevance. This may indicate that colostrum, cow feces, and calf feces harbor distinct microbial compositions, which could have implications for the maternal transfer of microbiota and its influence on neonatal gut development. It is plausible that with an increased sample size, this trend could achieve statistical significance, providing stronger evidence of the observed differences. Future studies with larger cohorts are warranted to confirm these findings and explore their implications further.

The taxonomic analysis showed that both the colostrum and feces of Hanwoo and Holstein cows and calves were dominated by Firmicutes, Proteobacteria, and Bacteroidetes. Similar studies have demonstrated Proteobacteria, Firmicutes, and Bacteroidetes as dominant phyla in the colostrum [19,41] and fecal [14,20] microbiota of both cows and calves. The study revealed a higher abundance of bacterial genera such as *Romboutsia*, *Paeniclostridium*, and *Lactobacillus* in Holstein cattle compared to Hanwoo cattle. Moreover, LEfSe identified differentially abundant genera between the Hanwoo and Holstein breeds, with the Holstein breed having higher relative abundances of genera such as *Clostridium* and *Mediterraneibacter*, while the Hanwoo breed showed enrichment in genera like *Mailhella* and *Aurantiacibacter*. This suggests that while the overall microbial diversity may not differ significantly between breeds, specific taxa might contribute differently to gut health and disease resistance in these cattle breeds. In addition, the study identified several genera that were differentially abundant in various sample types. For example, *Staphylococcus* and *Corynebacterium* were more abundant in the colostrum, while *Romboutsia*, *Paeniclostridium*, and other genera were predominant in cow feces. The calf fecal microbiota was notably enriched with *Lactobacillus* and *Limosilactobacillus*, which are known to be associated with gastrointestinal health in calves [41]. The differences in the microbial compositions of colostrum and feces are significant, as colostrum provides the initial microbial inoculum that shapes the neonatal gut microbiome [42]. Several studies have demonstrated bacterial communities that are distinct between different breeds of cattle and sample types [43,44,45]. The differences between the bacterial communities in different sample types from Holstein and Hanwoo cattle are likely influenced by variations in their management practices and the nutritional composition of their diets [43,46,47].

The detection of common genera between cow and calf microbiota across sample types highlights the potential vertical transmission of microbiota from the dam to the offspring. Moreover, the fact that colostrum shared more genera with calf feces compared to cow feces indicates that colostrum may play a pivotal role in shaping the early microbiota of calves [14,27,48,49]. Microbes present in the mother’s feces are thought to be the primary origin of neonates’ gastrointestinal microbiota [48,49]; however, vaginal microbiota may also be a potential source of microbes in neonates as differences were observed between infants born vaginally and by cesarean section [50,51,52]. The study also found that the microbial communities shared between colostrum and calf feces were higher in Holstein cattle compared to Hanwoo cattle, further reinforcing the idea that breed-specific microbial signatures exist and may influence calf health and productivity differently. *Prevotella*, *Bacteroides*, *Lactobacillus*, *Enterococcus*, *Clostridium*, *Bacillus*, *Bifidobacterium*, and *Ruminococcus* were among the genera shared between cow colostrum and calf fecal microbiota, as well as between the fecal microbiota of both cows and calves. The similarities between the microbiota of cows and calves suggest the possible occurrence of vertical microbial transfer. Furthermore, the presence of these genera indicates that colostrum serves as an early source for establishing the intestinal microbiota in newborn calves [46]. Similarly, several studies have also identified these genera in both maternal and calf fecal samples [14,17,53]. A study by Hang et al. [27] found that feeding colostrum soon after birth enhances the colonization of total bacteria in the gastrointestinal tract of calves within the first 12 h compared to calves not given colostrum. Moreover, the gut microbiota plays a crucial role in the health and development of calves, with disruption of the gut microbiome potentially leading to digestive diseases like diarrhea and inflammation [10]. The vertical transfer of microbial communities from the colostrum to calves is a significant factor in establishing the gut microbiota, which in turn influences calf health and productivity [11]. Further research is needed to fully understand the mechanisms and implications of this microbial transfer.

The findings of this study demonstrated distinct microbial profiles in colostrum, cow feces, and calf feces in both Holstein and Hanwoo cattle, highlighting the crucial role of maternal microbial transfer in shaping calf gut microbiota. Although no conclusive evidence of rotavirus transmission from dam to calf was identified, the findings highlight the complexity of microbial interactions and the importance of further research into potential transmission routes. Moreover, exploring the impact of external factors such as management practices and environmental conditions could offer a more comprehensive understanding of the dynamics of rotavirus transmission. The microbial similarities observed between the cow and calf samples reinforce the significant impact of maternal microbiota on calf gut health. Despite the overall similarity in bacterial diversity between the breeds, the specific microbial taxa enriched in each breed suggest that there are opportunities for breed-specific strategies to optimize gut health and calf productivity.

## 5. Conclusions

In conclusion, the study suggests that maternal microbiota transfer through colostrum significantly shapes the neonatal gut microbiota, with specific differences observed between colostrum and fecal samples from Holstein and Hanwoo cattle. Despite the detection of rotavirus in Holstein cattle, there was no definitive evidence of direct transmission from dam to calf, suggesting that other factors such as environmental contamination may play a role in rotavirus-associated diarrhea. The bacterial taxa identified in both the Holstein and Hanwoo breeds reveal potential microbial markers that may be associated with health and performance traits in each breed. Future research should expand the sample size and explore the long-term health effects of early microbial colonization, especially focusing on breed-specific differences to develop targeted interventions that enhance calf health and performance in dairy and beef production systems.

## Data Availability

The original contributions presented in the study are included in the article material, further inquiries can be directed to the corresponding author.
